# Impact of Childhood Environmental Unpredictability on Hoarding Behavior: Attachment as Mediator and Environmental Cues as Moderator

**DOI:** 10.1002/pchj.70057

**Published:** 2025-10-07

**Authors:** Hang Ma, Chengfang Wang, Ping Hu

**Affiliations:** ^1^ Department of Psychology Renmin University of China Beijing China

**Keywords:** attachment anxiety, attachment avoidance, childhood environmental unpredictability, environmental cues, hoarding behavior

## Abstract

This study examined the impact of childhood environmental unpredictability on hoarding behavior, focusing on the mediating roles of attachment anxiety and attachment avoidance and the moderating role of environmental cues. Three studies were conducted: Study 1 investigated the effect of childhood environmental unpredictability on hoarding behavior through big data analysis and an experiment; Study 2 tested the mediating effects of attachment anxiety and avoidance, as well as the moderating role of environmental cues, using a two‐stage questionnaire; and Study 3 further explored differences in hoarding behavior across attachment styles. Results indicated that childhood environmental unpredictability significantly and positively predicted hoarding behavior, with attachment anxiety and avoidance serving as parallel mediators. Moreover, pandemic‐related environmental cues moderated the direct effect of childhood environmental unpredictability on hoarding behavior, with this effect weakening after the cues diminished. These findings provide novel insights into hoarding behavior as an adaptive response to childhood environmental unpredictability, clarify the roles of attachment anxiety and avoidance as adaptive mechanisms, and underscore the influence of current environmental cues in shaping hoarding behavior.

## 1 Introduction

With rising material living standards, the concept of “decluttering” has received growing public attention, while hoarding behavior has become increasingly visible in social discourse. Importantly, hoarding behavior should not be equated with hoarding disorder (HD). Hoarding is common in non‐clinical populations and serves adaptive functions. From an evolutionary perspective, hoarding emerged as a survival strategy in response to environmental uncertainty. In ancestral hunter‐gatherer societies, the scarcity and unpredictability of resources made stockpiling food essential for survival. Even in modern contexts of material abundance, people display hoarding tendencies during crises, such as shortages caused by pandemic‐related lockdowns. This suggests that hoarding, as an adaptive resource‐acquisition strategy, remains embedded in daily life. However, existing research has largely centered on pathological hoarding—its etiology and interventions—while relatively little attention has been directed toward hoarding in the general population. To address this gap, the present study adopts an evolutionary psychology perspective, conceptualizing hoarding as an adaptive strategy for resource acquisition under environmental change and examining its underlying psychological mechanisms.

Research suggests that individuals who engage in hoarding behavior often have experienced childhood adversity, including material deprivation, exposure to crime, abuse, and dysfunctional parenting (Cromer et al. 2007; Landau et al. 2011), highlighting the potential role of early environmental factors in shaping hoarding behavior. Ellis et al. (2009) proposed a two‐dimensional model of environmental risk, which conceptualizes childhood environment in terms of two key dimensions: harshness and unpredictability. Compared to environmental harshness, unpredictability may have a more profound impact on development, as unpredictable stressors present greater challenges for adaptation (Ellis et al. 2009). While harsh yet stable environments pose consistent threats, their predictability allows individuals to develop coping strategies. In contrast, unpredictable environments, characterized by fluctuating resource availability and stress exposure, can undermine an individual's sense of control, potentially influencing behavior into adulthood (Mittal and Griskevicius 2014; Martinez et al. 2022).

Given the crucial role of childhood environmental unpredictability in shaping adult behavioral patterns, this study aims to explore the following research questions: (1) Does childhood environmental unpredictability, as a distal environmental factor, influence hoarding behavior? (2) If so, what are the underlying mechanisms? To address these questions, this study examines how childhood environmental unpredictability contributes to hoarding behavior, clarifies the mediating roles of attachment anxiety and attachment avoidance, and investigates the moderating role of environmental cues in this process.

### 1.1 Childhood Environmental Unpredictability and Hoarding Behavior

Life history theory posits that individuals' behavioral patterns are shaped by their childhood environment, particularly under conditions of environmental unpredictability (Mittal and Griskevicius 2014). Childhood environmental unpredictability refers to random fluctuations in disease prevalence and mortality rates within an individual's early surroundings (Ellis et al. 2009). Prior research has identified factors such as food resource instability, exposure to violence, inconsistent parenting practices, and frequent changes in living conditions as key indicators of childhood environmental unpredictability (Chang et al. 2019; Luo et al. 2020). Childhood environmental unpredictability signals instability in both interpersonal and material resources, fostering uncertainty about the future (Martinez et al. 2022). Such uncertainty may diminish an individual's sense of control and security (Brosschot et al. 2016). To cope with such insecurity, individuals may develop various compensatory psychological defense mechanisms, among which hoarding behavior may serve as an adaptive strategy to satisfy the need for psychological security. Research suggests that individuals attribute human‐like qualities to objects (i.e., anthropomorphism) and perceive them as extensions of their identity. Moreover, attachment to objects and their use serves as key sources of comfort and security (Frost and Hartl 1996; Yap and Grisham 2021). Additionally, heightened uncertainty about the future may amplify a perceived responsibility to prepare for potential scarcity, reinforcing the belief that objects have inherent functional utility. Consequently, individuals may be more inclined to acquire and retain items on a “just in case” basis (Frost and Gross 1993).

### 1.2 The Parallel Mediating Roles of Attachment Anxiety and Attachment Avoidance

Within the framework of life history theory, Chen (2017) introduced the attachment‐resource control theory, which posits that individuals' childhood environments and attachment experiences exert long‐term influences on their behavioral strategies in adulthood. Attachment functions as an evolved psychological mechanism that enables individuals to make strategic choices for survival and development (Chen 2017, 2018). Two key dimensions of attachment are attachment anxiety and attachment avoidance, both reflecting an individual's sense of insecurity within intimate relationships (Brennan et al. 1998). Individuals with high attachment anxiety fear abandonment and experience heightened insecurity, while those with high attachment avoidance prioritize independence and emotional distance (Collins and Read 1990).

Research indicates that greater childhood environmental unpredictability increases the likelihood of developing insecure attachment patterns, which in turn fosters acquisitive behaviors related to resource control (Chen 2018; Szepsenwol and Simpson 2019). Insecurely attached individuals tend to respond to interpersonal relationships with either avoidance (e.g., maintaining emotional distance) or anxiety (e.g., heightened vigilance and fear of abandonment) (Ainsworth et al. 2015). When faced with interpersonal rejection or relational instability, these individuals may redirect emotional attachment toward objects, perceiving them as stable, controllable, and reliable substitutes for human relationships (Steketee and Frost 2003; Yap et al. 2020). As a result, individuals with insecure attachment are more prone to developing emotional reliance on objects to compensate for unmet interpersonal security needs.

### 1.3 The Moderating Role of Environmental Cues

Environmental cues refer to various environmental stimuli or signals that trigger and guide specific behaviors or responses in individuals (Lindenberg 2018; Stämpfli et al. 2017). Exposure to environmental cues activates behavioral patterns shaped by both evolutionary and developmental processes. In research on the development of individual behavioral and psychological patterns in response to environmental adaptation, Griskevicius et al. (2013) introduced the sensitization model. Grounded in life history theory, this model proposes that early life experiences, such as resource scarcity and family discord, profoundly shape an individual's psychological and behavioral patterns. These formative experiences not only shape an individual's worldview and self‐perception but also play a crucial role in determining coping strategies in adulthood. The model further highlights that life‐history strategies exhibit substantial individual differences in response to environmental cues, with these variations rooted in early life experiences (Mittal et al. 2015). That is, life‐history strategies do not remain consistent across all situations but become pronounced specifically in the face of adversity or stress. For instance, Griskevicius et al. (2011) found that individuals from diverse childhood environments exhibited significant variations in reproductive timing strategies after exposure to mortality cues.

Hoarding behavior, an adaptive response shaped by early childhood experiences, personal traits, and environmental influences, aligns with the sensitization model, wherein environmental cues influence individuals' hoarding behavior (Wang and Hao 2020). Moreover, individuals from distinct childhood environments may exhibit varying object‐related response strategies under different environmental cues (Schumacher and Micheli 2024). In contexts of resource scarcity or perceived threats, individuals who experienced high levels of childhood environmental unpredictability may exhibit heightened hoarding behavior as a strategy to cope with anticipated resource uncertainty and deprivation. In contrast, those from more predictable childhood environments may not demonstrate the same hoarding tendencies.

### 1.4 The Present Study

The present study aims to examine the potential mediators (attachment anxiety and attachment avoidance) and the moderator (environmental cues) in the relationship between childhood environmental unpredictability and hoarding behavior. Specifically, this study constructed a moderated parallel mediation model to test the following hypotheses:

Hypothesis 1 *Childhood environmental unpredictability positively predicts hoarding behavior*.

Hypothesis 2 *Attachment anxiety and attachment avoidance serve as parallel mediators in the relationship between childhood environmental unpredictability and hoarding behavior*.

Hypothesis 3 *Environmental cues moderate the relationship between childhood environmental unpredictability and hoarding behavior. Specifically, individuals with different levels of childhood environmental unpredictability exhibit varying degrees of hoarding behavior when exposed to highly unpredictable environmental cues*.

**FIGURE 1:**
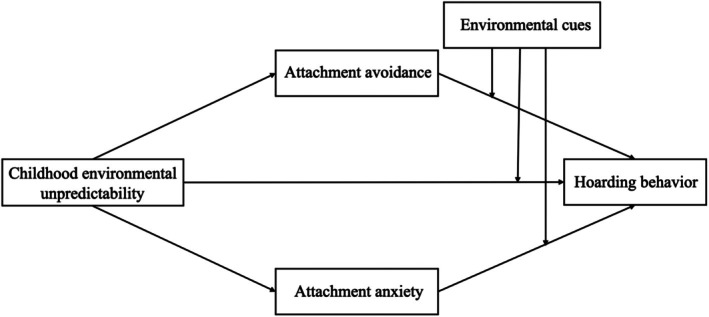
The theoretical model of this study.

The theoretical model is outlined in Figure 1.

## 2 Study 1a

### 2.1 Method

#### 2.1.1 Sample

The sample for this study, based on big data analysis, was collected from Zhihu, the largest knowledge‐sharing platform in China. Zhihu hosts an active and diverse user base, including industry experts, students, and professionals, making it a rich source of varied data. Additionally, its real‐time data dynamics enable research findings to capture and reflect contemporary societal trends.

#### 2.1.2 Procedure and Measures

On digital content platforms, users' hoarding behaviors are primarily manifested through the bookmarking of substantial amounts of material or content in their favorites (Wang et al. 2023; Wu et al. 2024). Consequently, a larger collection volume in Zhihu users' data indicates a greater likelihood of hoarding. By using Zhihu's search function, we can locate relevant questions that match specific keywords. For instance, based on the Early Life Environmental Unpredictability Scale revised by Luo et al. (2020), childhood environmental unpredictability includes both residential and parental emotional unpredictability. Searching for “frequent relocations in childhood” enabled us to find a list of related questions, and by collecting user data from these questions, we obtained data regarding the unpredictability of the childhood environment. Similarly, searching for keywords such as “always in the same place during childhood” allowed us to obtain data from users with predictable childhood environments. In the current study, based on the widely used childhood environmental unpredictability scale, we selected several keyword sets to collect relevant data (see Table 1).

**TABLE 1:** Relevant variables and search keywords in Study 1a.

Variable	Tendency	Keywords
Childhood environmental unpredictability	Positive (Unpredictable)	Frequent relocations in childhood
		Parental divorce in childhood
		Parental death in childhood
		Family upheaval in childhood
		Childhood instability
	Negative (predictable)	Stable residence during childhood
		Stable family environment in childhood
		Emotional stability of parents in childhood
		Happy childhood experience
		Positive childhood experience

After searching for keywords, the study retrieved a list of relevant questions using the Node.js Puppeteer crawling technique. Due to the search and recommendation algorithms of the Zhihu platform, the list of questions retrieved from the search may not be entirely relevant to the variables. GPT‐4, a generative multimodal pre‐trained model, is widely used in semantic analysis (Che et al. 2023). Therefore, the study utilized GPT‐4 to score the degree of association between question descriptions and variables, thereby excluding questions that were not relevant to the variables. The study enhanced GPT‐4's performance in the relevance‐scoring task through the design of input prompts. Specifically, the study constructed a set of predefined criteria and examples to assist the model in more accurately understanding the core meaning of the variables and their association with the problem description. For example, the model was provided with core definitions of the variables and a sample question with a strong keyword match. Finally, the study once again collected data from all users who provided answers, using crawling technology to save their favorite data, thereby obtaining each user's tendency and collection volume. The final collected data for the questions complies with Zhihu's User Agreement for public content and has been de‐identified.

SPSS 25.0 was used to analyze the relationship between childhood environmental unpredictability and hoarding behavior by examining users' tendencies and collection volume. The independent samples *t*‐test was used to compare the mean collection volume between two groups. The collection volume served as the test variable, while the tendency of childhood environmental unpredictability was used as the grouping variable to examine whether the results were statistically significant. The tendencies were quantified by assigning a value of 0 to positive tendencies and a value of 1 to negative tendencies.

### 2.2 Results

The dataset comprised 44,844 users, with 13,771 categorized into the predictable childhood environment group and 31,073 into the unpredictable childhood environment group. The independent samples *t*‐test indicated that users from unpredictable childhood environments (*M* = 116.65, SD = 613.76) exhibited significantly higher collection volumes than those from predictable childhood environments (*M* = 104.23, SD = 612.03), *t* = −1.98, *p* < 0.05, Cohen's *d* = 0.02. Although statistically significant, the effect size was negligible. One explanation is that the number of collection volumes, while serving as a digital proxy for hoarding on content platforms, does not fully capture hoarding behavior. Moreover, it is easily influenced by factors such as platform usage habits, recommendation algorithms, and other non‐psychological variables. Therefore, this result should be considered exploratory, offering preliminary support for subsequent studies. To further establish causality, Study 1b employed a text priming paradigm to manipulate environmental unpredictability and assess its impact on hoarding behavior.

## 3 Study 1b

### 3.1 Method

#### 3.1.1 Participants

This study used a convenience sampling method to recruit participants from Renmin University of China. Recruitment information was disseminated through social media platforms. Based on calculations using G*Power (Faul et al. 2007), a sample size of 128 participants was required to achieve a statistical power of 1 − *β* = 0.80 for a medium effect size (*t*‐test, *d* = 0.50). A total of 137 participants were recruited, including 97 males and 40 females. The sample comprised 130 undergraduates, six master's students, and one doctoral student; 75 participants were from urban areas and 62 from rural areas; 65 were only children and 72 were non‐only children. The mean age was 21.57 years (SD = 2.37).

#### 3.1.2 Procedures

A between‐subjects experimental design with a single factor (environmental unpredictability: high vs. low) was employed. Participants were randomly assigned to either the high environmental unpredictability group (*n* = 73) or the low environmental unpredictability group (*n* = 64). Following the experimental procedure of Griskevicius et al. (2011), each group completed a text‐based priming task. Participants in the high unpredictability group read a fabricated news article describing a severe national employment crisis, emphasizing environmental instability. In contrast, the low unpredictability group read an article of identical format and length, but depicting a stable, positive national situation. All participants were informed that the articles were sourced from an authoritative Chinese government media outlet (Xinhua News Agency) and were told the experiment was a memory test, requiring them to recall and summarize the article content after reading. The research protocol was reviewed and approved by an independent ethics committee. Written informed consent was obtained from all participants prior to participation.

#### 3.1.3 Measures

The environmental unpredictability manipulation check followed the experimental paradigm of Griskevicius et al. (2011). After reading the provided materials, participants completed a questionnaire designed to assess their subjective perceptions of the environment. The questionnaire included five items rated on a 7‐point Likert scale (1 = *not at all*, 7 = *very much*), measuring perceptions of environmental danger, unpredictability, and emotional arousal. An example item is, “To what extent do you feel the environment has become more unpredictable?”

Hoarding beliefs were assessed using the Saving Beliefs Questionnaire (SBQ) developed by Timpano et al. (2015) and adapted for Chinese university students. The scale comprises seven dimensions: emotional attachment, memory, control, responsibility, wastefulness, usefulness, and aesthetic qualities, and was answered using a 7‐point Likert scale (1 = *not at all*, 7 = *very much*). The Cronbach's *α* coefficient of the scale in the study was 0.85.

### 3.2 Results

#### 3.2.1 Manipulation Check

An independent samples *t*‐test was conducted to examine differences between the two groups on five items. Results indicated no significant difference in emotional arousal between the high unpredictability group (*M* = 5.65, SD = 1.35) and the low unpredictability group (*M* = 5.56, SD = 0.89), *t* = −0.49, *p* = 0.623, Cohen's *d* = −0.08. However, the high unpredictability group (*M* = 5.48, SD = 1.08) scored significantly higher than the low unpredictability group (*M* = 3.47, SD = 1.49) on items 1–4, which assessed environmental danger and unpredictability, *t* = −8.88, *p* < 0.001, Cohen's *d* = −1.56. These results confirm the effectiveness of the experimental manipulation.

#### 3.2.2 Environmental Unpredictability and Hoarding Behavior

The results of the independent samples *t*‐test indicated that participants in the high unpredictability group (*M* = 5.31, SD = 0.65) exhibited significantly stronger hoarding beliefs compared to those in the low unpredictability group (*M* = 4.85, SD = 0.75), *t* = −3.92, *p* < 0.001, Cohen's *d* = −0.66. The experimental results indicated that participants in the high environmental unpredictability condition exhibited significantly stronger hoarding beliefs compared to those in the low environmental unpredictability condition. This result further supports the findings from Study 1a and provides causal evidence that environmental unpredictability may contribute to individuals' hoarding tendencies. Building on this, Study 2 employed a two‐stage questionnaire to further investigate the impact of childhood environmental unpredictability on hoarding behavior and its underlying mechanisms.

## 4 Study 2

### 4.1 Method

#### 4.1.1 Participants

This study employed convenience sampling, and data were collected in two phases. The first phase took place amid the COVID‐19 pandemic (July–September 2022), yielding 662 valid responses. The second phase occurred after adjustments to pandemic control policies (February–August 2023), yielding 749 valid responses. The survey was administered via the Credamo online platform. All participants provided electronic informed consent before participation, which ensured voluntary involvement and the right to withdraw at any time. To ensure data quality, fixed‐response items (attention checks) were included, and responses were excluded if the consent form was missing, response patterns were uniform, or completion time was unreasonably short. After these procedures, 1411 valid responses were retained.

The final sample included 665 males and 746 females, with a mean age of 22.31 years (SD = 4.52). In terms of educational attainment, 81 participants had less than a bachelor's degree, 1137 held a bachelor's degree, 167 a master's degree, and 26 a doctoral degree. Among them, 738 participants reported an urban background and 673 a rural background; 861 were only children, whereas 550 were not.

#### 4.1.2 Measures

Childhood environmental unpredictability was assessed using the Early Environmental Unpredictability Scale, revised by Luo et al. (2020). This scale comprises nine items, including three items measuring residential environmental unpredictability and six items assessing parental emotional unpredictability. Participants responded using a 7‐point Likert scale (1 = *not at all consistent*, 7 = *completely consistent*). The Cronbach's *α* coefficient for this scale in the current study was 0.86.

Attachment anxiety and attachment avoidance were assessed using the Experiences in Close Relationships Inventory (ECR), revised by Li and Kato (2006), originally developed by Brennan et al. (1998). The scale consists of two dimensions: attachment avoidance (18 items) and attachment anxiety (18 items). Participants responded using a 7‐point Likert scale (1 = *strongly disagree*, 7 = *strongly agree*). In the present study, the Cronbach's *α* coefficient was 0.85 for attachment avoidance and 0.92 for attachment anxiety.

Hoarding behavior was assessed using the revised version of Frost et al.'s (2004) Saving Inventory‐Revised (SI‐R), adapted for Chinese college students by Tang et al. (2012). The scale consists of two factors: excessive acquisition–difficulty discarding and clutter. The SI‐R contains 21 items, and participants responded using a 5‐point Likert scale (0 = *very little*; 4 = *almost all or completely*). In this study, the Cronbach's *α* coefficient was 0.96.

In this study, the COVID‐19 pandemic was used as an environmental cue to enhance the ecological validity of the research by examining changes in individual hoarding behavior in response to the pandemic. Dummy variables were assigned to represent the environmental cues, with questionnaires collected during the pandemic coded as 0 and those collected after the pandemic coded as 1.

### 4.2 Results

#### 4.2.1 Common Method Bias Test

Harman's single‐factor test was conducted to test common‐method bias. The results revealed that nine factors had eigenvalues greater than 1. The first factor explained only 28.75% of the variance, which was below the standard threshold of 40% (Tang and Wen 2020), indicating no severe common‐method bias in this study.

#### 4.2.2 Descriptive Statistics

Descriptive statistics and correlation results are presented in Table 2. The results indicated significant positive correlations between childhood environmental unpredictability, attachment avoidance, attachment anxiety, and hoarding behavior. Additionally, gender was significantly associated with childhood environmental unpredictability, attachment avoidance, attachment anxiety, and hoarding behavior. Moreover, age exhibited a significant negative correlation with attachment avoidance and attachment anxiety.

**TABLE 2:** Descriptive statistics and correlation matrix (*N* = 1411).

Variable	*M*	SD	1	2	3	4	5	6
1. Childhood environmental unpredictability	2.66	0.85	1.00					
2. Attachment avoidance	3.34	0.89	0.40**	1.00				
3. Attachment anxiety	3.98	1.16	0.49**	0.38**	1.00			
4. Hoarding behavior	1.62	0.86	0.47**	0.29**	0.53**	1.00		
5. Gender (0 = M; 1 = F)	0.47	0.50	0.07**	−0.08**	0.11**	0.15**	1.00	
6. Age	22.31	4.52	0.05	−0.08**	−0.13**	0.01	0.03	1.00

*Note*: **p < 0.01.

#### 4.2.3 Parallel Mediation Analysis of Attachment Avoidance and Attachment Anxiety

Mediation analyses were conducted using Model 4 in the Process v3.4.1 plugin for SPSS. Controlling for gender and age, the parallel mediating effects of attachment avoidance and attachment anxiety in the relationship between childhood environmental unpredictability and hoarding behavior were examined. All continuous variables were mean‐centered, and standardized coefficients were used in the analysis. As shown in Table 3, childhood environmental unpredictability significantly predicted hoarding behavior (*β* = 0.46, *t* = 19.61, *p* < 0.001). When the mediating variables—attachment avoidance and attachment anxiety—were included, childhood environmental unpredictability remained a significant predictor of hoarding behavior (*β* = 0.25, *t* = 9.81, *p* < 0.001). Childhood environmental unpredictability significantly predicted attachment avoidance (*β* = 0.41, *t* = 16.88, *p* < 0.001) and attachment anxiety (*β* = 0.49, *t* = 21.20, *p* < 0.001). In addition, attachment avoidance significantly predicted hoarding behavior (*β* = 0.06, *t* = 2.31, *p* < 0.05), while attachment anxiety also significantly predicted hoarding behavior (*β* = 0.38, *t* = 14.57, *p* < 0.001).

**TABLE 3:** The mediating effects test of attachment avoidance and attachment anxiety.

Regression equation (*N* = 1411)		Fit metrics			Coefficient significance	
Outcome variable	Predictor variable	*R* ^*2*^	Δ*R* ^*2*^	*F*	*β* (SE)	*t*
HB	CEU	0.23	0.23	141.22***	0.46 (0.02)	19.61***
AA	CEU	0.18	0.18	101.89***	0.41 (0.03)	16.88***
AX	CEU	0.26	0.26	168.36***	0.49 (0.03)	21.20***
HB	CEU	0.35	0.12	148.90***	0.25 (0.03)	9.81***
	AA				0.06 (0.02)	2.31*
	AX				0.38 (0.02)	14.57***

*Note*: *p < 0.05, ***p < 0.001; All variables were standardized and brought into the regression equation.

Abbreviations: CEU, childhood environmental unpredictability; HB, hoarding behavior; AA, attachment avoidance; AX, attachment anxiety.

The results of the mediation analysis are presented in Table 4. Childhood environmental unpredictability exerted a significant direct effect on hoarding behavior (direct effect = 0.26, 95% CI [0.21, 0.31]). In addition, significant indirect effects were observed through both attachment avoidance (indirect effect = 0.02, 95% CI [0.002, 0.05]) and attachment anxiety (indirect effect = 0.19, 95% CI [0.15, 0.22]). These findings suggest that childhood environmental unpredictability influences hoarding behavior both directly and indirectly via attachment‐related mechanisms, with attachment anxiety exerting a stronger mediating effect than attachment avoidance.

**TABLE 4:** Mediation effect decomposition.

Effect	Effect value	Proportion (relative to total effect %)	BootLLCI	BootULCI
Total effect	0.47		0.42	0.51
Direct effect	0.26	55.1	0.21	0.31
Indirect effect (total)	0.21	44.9	0.17	0.25
Indirect effect (attachment avoidance)	0.02	5.0	0.002	0.05
Indirect effect (attachment anxiety)	0.19	39.9	0.15	0.22

**FIGURE 2:**
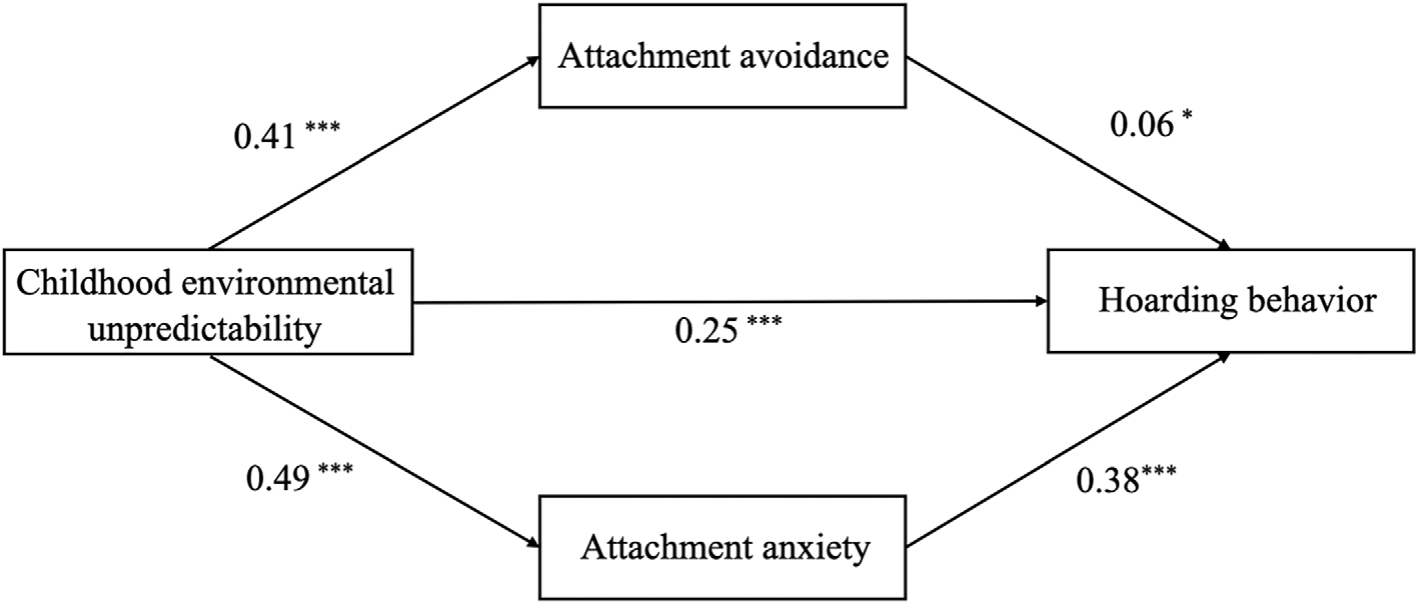
Parallel mediation model of attachment anxiety and attachment avoidance. Standardized regression coefficients are displayed for all paths. *p < 0.05; ***p < 0.001.

The parallel mediation model of attachment avoidance and attachment anxiety is depicted in Figure 2.

#### 4.2.4 Moderated Mediation Model Test

Moderated mediation analyses were performed using Model 15 in the Process v3.4.1 plugin for SPSS. After controlling for gender and age, the study examined the moderating role of pandemic‐related environmental cues, with childhood environmental unpredictability as the independent variable, hoarding behavior as the dependent variable, and attachment avoidance and attachment anxiety as mediators. Pandemic‐related environmental cues were coded as a dummy variable (0 = *mid‐pandemic*, 1 = *post‐pandemic*). All continuous variables were mean‐centered prior to analysis. As shown in Table 5, childhood environmental unpredictability was a significant predictor of hoarding behavior (*β* = 0.35, *t* = 9.08, *p* < 0.001). Both attachment avoidance (*β* = 0.08, *t* = 2.33, *p* < 0.05) and attachment anxiety (*β* = 0.28, *t* = 10.18, *p* < 0.001) significantly predicted hoarding behavior. Furthermore, environmental cues had a significant effect on hoarding behavior (*β* = 0.15, *t* = 4.19, *p* < 0.001). The interaction between environmental cues and childhood environmental unpredictability was significant in predicting hoarding behavior (*β* = −0.16, *t* = −3.07, *p* < 0.01). However, the interaction effect between environmental cues and attachment avoidance was not significant (*β* = −0.04, *t* = −0.88, *p* = 0.377), nor was the interaction effect between environmental cues and attachment anxiety (*β* = −0.005, *t* = −0.13, *p* = 0.897). These findings suggest that environmental cues moderated the direct relationship between childhood environmental unpredictability and hoarding behavior.

**TABLE 5:** Moderated mediation model test.

Regression equation (*N* = 1411)		Fit metrics			Coefficient significance	
Outcome variable	Predictor variable	*R* ^*2*^	Δ*R* ^*2*^	*F*	*β* (SE)	*t*
Hoarding behavior	Childhood environmental unpredictability	0.35	0.35	128.27***	0.26 (0.03)	10.14***
	Attachment avoidance				0.06 (0.02)	2.36*
	Attachment anxiety				0.28 (0.02)	14.76***
	Environmental cues				0.15 (0.04)	4.09***
Hoarding behavior	Childhood environmental unpredictability	0.36	0.01	88.38***	0.35 (0.04)	9.08***
	Attachment avoidance				0.08 (0.03)	2.33*
	Attachment anxiety				0.28 (0.03)	10.18***
	Environmental cues				0.15 (0.04)	4.19***
	Int_1				−0.16 (0.05)	−3.07**
	Int_2				−0.04 (0.05)	−0.88
	Int_3				−0.005 (0.04)	−0.13

*Note*: *p < 0.05, **p < 0.01, ***p < 0.001; Int_1 = childhood environmental unpredictability × environmental cues, Int_2 = attachment avoidance × environmental cues, Int_3 = attachment anxiety × environmental cues.

**FIGURE 3:**
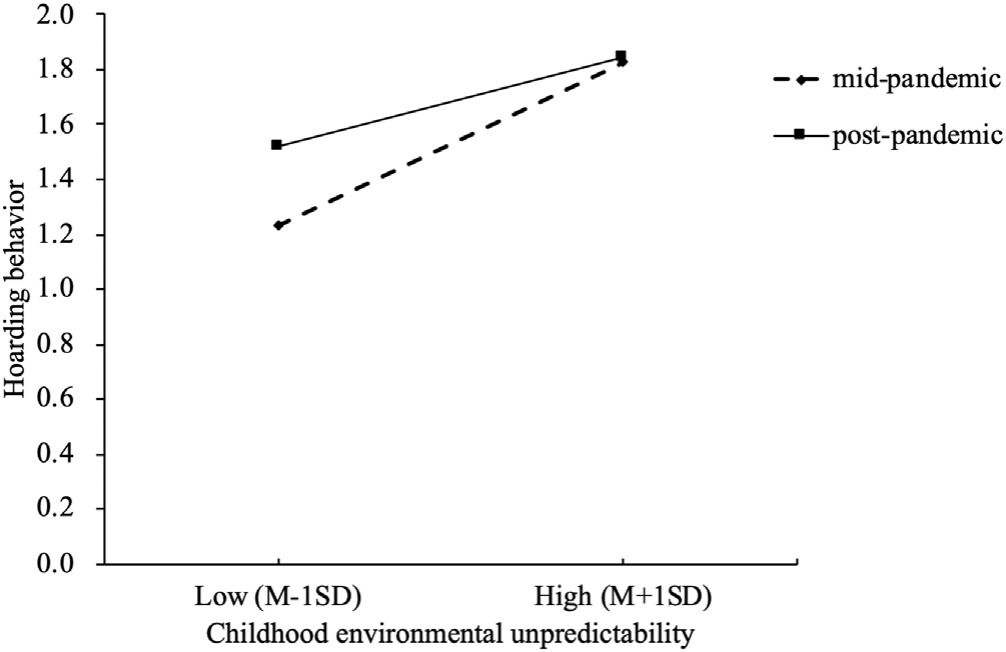
Simple slope analysis of the interaction between childhood environmental unpredictability and pandemic‐related environmental cues on hoarding behavior.

Simple slope analyses further revealed that childhood environmental unpredictability was a significant positive predictor of hoarding behavior during the COVID‐19 pandemic (coded as 0; *β* = 0.35, *t* = 9.08, *p* < 0.001). However, in the post‐pandemic period (coded as 1), although childhood environmental unpredictability remained a significant positive predictor of hoarding behavior, its effect was weakened (*β* = 0.19, *t* = 5.58, *p* < 0.001) (see Figure 3). Additionally, the results revealed that the difference in hoarding behavior between individuals with high and low childhood environmental unpredictability was more pronounced during the pandemic than in the post‐pandemic period. Study 2 replicated the findings from Study 1a and Study 1b and conducted a preliminary examination of the mediating roles of attachment anxiety and attachment avoidance, as well as the moderating effect of environmental cues, at the correlational level. Building on this foundation, Study 3 further explored differences in hoarding behaviors among individuals with distinct attachment types.

## 5 Study 3

### 5.1 Method

#### 5.1.1 Participants

This study recruited 423 college students via the Credamo online platform. To ensure data quality, responses were excluded if participants declined to complete the questionnaire, failed attention checks, exhibited uniform response patterns, or completed the survey in an unreasonably short time. After these procedures, 400 valid responses were retained, yielding a response rate of 94.6%. The final sample comprised 162 males and 238 females, with a mean age of 20.95 years (SD = 2.22). Regarding current enrollment, 288 participants were undergraduates, 84 were master's students, and 28 were doctoral students. Additionally, 269 participants reported an urban background and 131 a rural background; 230 were only children and 170 were not.

#### 5.1.2 Measures

Attachment avoidance and attachment anxiety were assessed using the same measurement tool employed in Study 2. Participants responded on a 7‐point Likert scale (1 = *strongly disagree*, 7 = *strongly agree*). In this study, Cronbach's *α* coefficient for the attachment avoidance dimension was 0.86, and for the attachment anxiety dimension, it was 0.92.

The measurement tool for hoarding behavior was also consistent with Study 2. Participants responded using a 5‐point Likert scale (0 = *very little*; 4 = *almost all or completely*). In this study, Cronbach's *α* coefficient for the scale was 0.96.

### 5.2 Results

To categorize participants' attachment types, this study employed *K*‐means clustering based on attachment anxiety and attachment avoidance, grouping them into four attachment types (Bartholomew and Horowitz 1991): secure, characterized by low levels of both attachment anxiety and attachment avoidance; preoccupied, with high attachment anxiety and low attachment avoidance; dismissing, with low attachment anxiety and high attachment avoidance; and fearful, characterized by high levels of both attachment anxiety and attachment avoidance. The distribution of these attachment types among participants was 26.5%, 14.5%, 30.5%, and 28.5%, respectively, as shown in Table 6.

**TABLE 6:** A cluster analysis of attachment styles.

Type	Secure (*n* = 106)		Preoccupied (*n* = 58)		Dismissing (*n* = 122)		Fearful (*n* = 114)		*F*	*η* ^2^
	*M*	SD	*M*	SD	*M*	SD	*M*	SD		
Attachment anxiety	2.75	0.64	4.55	0.64	3.81	0.64	5.23	0.54	315.22***	0.71
Attachment avoidance	2.72	0.52	2.47	0.51	4.17	0.58	3.90	0.47	240.31***	0.65

*Note*: ***p < 0.001.

A one‐way ANOVA was conducted, with attachment type as the independent variable and hoarding behavior as the dependent variable. The results indicated significant differences in hoarding behavior across different attachment types (*F* (3, 396) = 63.60, *p* < 0.001, *η*_*p*_^2^ = 0.33). Post hoc tests revealed that hoarding behavior scores followed this pattern: secure (*M* = 0.95, SD = 0.64) < preoccupied (*M* = 1.43, SD = 0.79) < dismissing (*M* = 1.57, SD = 0.62) < fearful (*M* = 2.27, SD = 0.83). Moreover, individuals with preoccupied, dismissing, and fearful attachment exhibited significantly higher hoarding behavior than those with secure attachment (*p* < 0.001), and fearful attachment individuals showed significantly higher hoarding behavior than both preoccupied and dismissing attachment individuals (*p* < 0.001). No significant difference was found between preoccupied and dismissing attachment types (*p* = 0.846). These results demonstrate significant differences in hoarding behaviors among individuals with varying attachment styles, further highlighting the crucial role of attachment in the relationship between childhood environmental unpredictability and hoarding behavior.

## 6 Discussion

Based on life history theory, this study explored hoarding behavior from an evolutionary perspective, proposing that it may represent an adaptive response to early childhood environmental conditions. Furthermore, drawing on the cognitive‐behavioral model of hoarding, this study examined the parallel mediating roles of attachment avoidance and attachment anxiety. Finally, integrating the sensitization model, this study investigated how distal and proximal environmental cues jointly shape hoarding behavior.

### 6.1 Childhood Environmental Unpredictability and Hoarding Behavior

This study found that childhood environmental unpredictability directly predicted hoarding behavior. Early environmental unpredictability shapes an individual's cognitive schema, fostering “uncertain perceptions” of the world—a belief that the external environment is volatile and relationships are unreliable (Szepsenwol and Simpson 2019). Moreover, individuals may extrapolate future scenarios from early life experiences, adjusting their behavioral strategies to maximize adaptive advantages in unpredictable environments (Belsky 2012). Childhood environmental unpredictability thus influences cognitive and behavioral patterns, leading individuals to prioritize rapid resource acquisition strategies in unstable environments to enhance their sense of security and ensure survival (Maranges et al. 2022). In this context, hoarding behavior emerges as an adaptive response, enabling individuals to acquire and safeguard resources in the face of environmental uncertainty. Therefore, from an evolutionary perspective, hoarding behavior should not be solely viewed as a maladaptive or pathological response. Instead, it may function as an adaptive strategy aimed at mitigating environmental unpredictability, promoting resource security, and ultimately enhancing survival and well‐being (Ma et al. 2024).

### 6.2 Parallel Mediating Roles of Attachment Avoidance and Attachment Anxiety

The present study found that attachment avoidance and attachment anxiety served as parallel mediators in the relationship between childhood environmental unpredictability and hoarding behavior. Specifically, childhood environmental unpredictability increased individuals' levels of attachment anxiety and attachment avoidance, which in turn exacerbated hoarding behavior. Attachment systems continue to shape cognition, emotion, and behavior throughout an individual's life, playing a crucial role in evolutionary adaptation. Different attachment styles function as mediators in specific contexts, influencing individuals' adaptive responses. While insecure attachment may not be inherently positive or desirable, it can still provide greater adaptive value in unpredictable environments, even surpassing secure attachment (Szepsenwol and Simpson 2019). Under this adaptive attachment pattern, individuals with high childhood environmental unpredictability may transfer emotional attachment to objects, leading to hoarding behavior. According to the cognitive‐behavioral model of hoarding, early adverse experiences shape individuals' beliefs about objects, fostering irrational attachment patterns. These attachment patterns, in turn, influence individuals' emotional responses and attitudes toward possessions, ultimately contributing to hoarding behavior (Frost and Hartl 1996).

Moreover, the mediating effect of attachment avoidance in the relationship between childhood environmental unpredictability and hoarding behavior was weaker than that of attachment anxiety. This disparity may arise because, although both attachment patterns are rooted in the unpredictability of early environments, they manifest in distinct ways. Driven by secondary attachment strategies, attachment‐anxious individuals typically employ hyperactivating strategies, striving to maintain proximity to their attachment figures and constantly monitoring emotional fluctuations to prevent weakening of the emotional bond. In contrast, attachment‐avoidant individuals use deactivating strategies, suppressing attachment needs, prioritizing independence, and denying emotional dependency (Mikulincer and Shaver 2003, 2007). Consequently, while individuals with both attachment styles may redirect their emotional attachment from interpersonal relationships to objects to compensate for a lack of security, attachment‐avoidant individuals are more likely to rely on themselves in both interpersonal and material contexts, resulting in relatively lower levels of hoarding (Danet and Secouet 2018).

### 6.3 Moderating Role of Environmental Cues

The present study investigated the moderating role of environmental cues in the relationships among childhood environmental unpredictability, attachment avoidance, attachment anxiety, and hoarding behavior using a two‐stage questionnaire. The results revealed that pandemic‐related environmental cues moderated the direct effect of childhood environmental unpredictability on hoarding behavior, further supporting the sensitization model. Moreover, hoarding behavior increased after the pandemic among individuals with both high and low levels of childhood environmental unpredictability. This finding underscores the significant influence of current environmental cues and challenges the notion of “innate determinism,” suggesting that individual behavior results from the interplay between early and current environments. Accordingly, when behavioral issues such as hoarding arise, they should not be attributed solely to an immutable childhood environment. Instead, individuals are encouraged to cultivate a positive and stable current environment to mitigate the emergence and escalation of problematic behaviors. Furthermore, while environmental cues influence behavioral choices, the extent of their impact depends on individuals' childhood environments, highlighting the importance of childhood environmental stability in shaping individual development. Consequently, parents should foster a stable living and emotional environment to facilitate the development of a secure internal working model in their children, enabling them to adapt more effectively to external environmental changes.

Finally, this study did not find a moderating effect of environmental cues on the relationships between attachment anxiety and hoarding behavior or between attachment avoidance and hoarding behavior. This may be due to the stability of adult attachment patterns. Individuals develop specific attachment schemas through interactions with their primary caregivers, and these cognitive frameworks typically remain relatively stable throughout adolescence and adulthood, continuously shaping their interpersonal interactions and interpretations of social contexts (Bretherton 1992; Scharfe and Bartholomew 1994). Therefore, pandemic‐related environmental cues may not be sufficient to significantly alter individuals' attachment patterns, thus failing to moderate the relationship between attachment and hoarding behavior.

### 6.4 Practical Implications

The findings of this study provide potential implications for the intervention of hoarding behavior. Attachment was found to mediate the association between childhood environmental unpredictability and hoarding behavior, suggesting that interventions should focus on addressing early attachment vulnerabilities to reduce the long‐term impact of adverse childhood experiences. Treatment may involve processing and repairing attachment‐related trauma to foster secure attachment, while family involvement can enhance caregivers' understanding of the traumatic roots of hoarding and strengthen individuals' sense of safety (Cohen et al. 2011). In addition, as proximal environmental cues such as the COVID‐19 pandemic were shown to amplify the link between childhood environmental unpredictability and hoarding behavior, intervention and prevention programs may benefit from incorporating environmental cue management strategies, such as psychoeducation to reduce excessive sensitivity to threat cues.

### 6.5 Limitations and Future Directions

This study employed big data analysis, an experimental approach, and survey research to examine the impact of childhood environmental unpredictability on hoarding behavior, as well as the roles of attachment and environmental cues. While offering practical implications, the study also has certain limitations.

First, Study 1a conducted an initial exploration of the relationship between childhood environmental unpredictability and hoarding behavior through big data analysis of a question‐and‐answer website. While big data analysis can capture diverse behavioral patterns, online user behavior may be shaped by external factors—such as platform algorithms and sociocultural influences—rather than solely reflecting an individual's childhood environment. Meanwhile, as childhood environmental unpredictability is a distal factor that is challenging to manipulate directly in experiments, Study 1b investigated its effects by experimentally manipulating short‐term environmental cues. However, such short‐term manipulation may not fully replicate the prolonged uncertainty of childhood environments. Furthermore, research suggests that hoarding behavior may emerge in childhood or adolescence and progressively intensify with age (Zaboski et al. 2019). Therefore, future research could employ a longitudinal design to track the developmental trajectory of hoarding behavior, offering a more comprehensive understanding of the long‐term effects of childhood environmental unpredictability on hoarding behavior.

Second, Study 2 used the COVID‐19 pandemic as an environmental cue to examine its role as a moderator, enhancing the ecological validity of the study. However, as a long‐term and pervasive environmental factor, the impact of the pandemic may be subject to multiple external influences, thus limiting causal inference. Future research could employ more precise laboratory‐based manipulations of environmental cues and control for potential confounding variables to further validate the role of environmental cues.

Finally, this study has several limitations related to its sample. First, the participants were exclusively from China. Previous research indicates that cultural factors, particularly collectivism versus individualism, serve as a crucial framework for shaping attachment patterns and their psychological consequences (Chi Kuan Mak et al. 2010). As a prototypical collectivist society, China emphasizes familial bonds and places high importance on parent–child relationships, which may make individuals more sensitive to relational threats when exposed to unpredictable childhood environments, thereby increasing susceptibility to insecure attachment (Frías et al. 2014). Insecure attachment may also lead to more adverse psychological outcomes in collectivist (Friedman et al. 2010). Moreover, China's long agrarian history has fostered cultural concepts such as “storing grain against famine,” “harvesting in autumn and storing in winter,” and “preparing for rainy days,” which may provide adaptive meaning to hoarding behavior. Consequently, environmental cues such as the COVID‐19 pandemic may have particularly strong priming effects, reinforcing hoarding tendencies. Future research should therefore examine the universality and cultural specificity of the model across different cultural contexts. Second, the current study focused on nonclinical populations. While the findings provide valuable insights into hoarding behaviors in the general population, their generalizability to clinical populations with hoarding disorder remains uncertain. Future studies should replicate these findings in clinical samples to evaluate their broader applicability and relevance for treatment.

## Ethics Statement

The study complies with the ethical standards of the Institutional Review Board (IRB) of Renmin University of China. All participants provided informed consent prior to participation, in accordance with the approved protocol (IRB Approval Number: 23‐008).

## Conflicts of Interest

The authors declare no conflicts of interest.

## Data Availability

The data that support the findings of this study are available from the corresponding author upon reasonable request.
